# Determination of mitral valve area with echocardiography, using intra-operative 3-dimensional versus intra- & post-operative pressure half-time technique in mitral valve repair surgery

**DOI:** 10.1186/1749-8090-8-98

**Published:** 2013-04-17

**Authors:** Woon-Seok Kang, Jae Won Choi, Joo-Eun Kang, Jin Woo Chung, Seong-Hyop Kim

**Affiliations:** 1 Department of Anaesthesiology and Pain medicine, Konkuk University Hospital, Konkuk University Medical Center, Konkuk University School of Medicine, Seoul, South Korea; 2 Research Institute of Medical Science, Konkuk University School of Medicine, Seoul, South Korea; 3 Department of Thoracic and Cardiovascular Surgery, Konkuk University Hospital, Konkuk University Medical Center, Konkuk University School of Medicine, Seoul, South Korea

**Keywords:** Mitral valve repair, 3D echocardiography, Pressure half-time

## Abstract

**Background:**

We hypothesized that mitral valve areas (MVAs) with echocardiography, using 3D planimetry technique (measured at one point at maximal opening of mitral valve) versus pressure half-time technique (PHT, measured during entire diastolic phase) in mitral valve repair surgery (MVR) would be different.

**Methods:**

Patients who had undergone MVR were retrospectively reviewed, and two different observers measured the MVAs using PHT and 3D planimetry technique. The MVAs derived from recorded medical data, using PHT and 3D planimetry technique were abbreviated to MVA-PHT1 and MVA-3D1, and data from the PHT and 3D planimetry techniques by observer A and observer B were determined as MVA-PHT2 and MVA-3D2, and MVA-PHT3 and MVA-3D3, respectively. The MVA derived by post-operative transthoracic echocardiography using the PHT technique was determined as MVA-TTE.

**Results:**

Intraclass correlation coefficients were 0.90 for the intra-operative PHT technique and 0.78 for the intra-operative 3D planimetry technique. MVA-3D1 (2.91 ± 0.65 cm^2^), MVA-3D2 (3.00 ± 0.63 cm2) and MVA-3D3 (2.97 ± 0.88 cm^2^) were significantly larger than MVA-TTE (2.40 ± 0.59 cm^2^), but intra-operative MVAs-PHT were not. The biases and precisions were larger, and the correlation coefficients were lower in 3D planimetry technique compared with PHT technique.

**Conclusions:**

MVA measured by 3D planimetry technique with TEE at the intra-operative post-MVR period was seemed to be larger than that measured by the PHT technique with TTE at the post-operative period. However, it did not mean that the 3D planimetry technique was inaccurate but needs cautions at determination of MVA using different techniques.

## Background

Determination of the mitral valve area (MVA) with intra-operative transoesophageal echocardiography (TEE) is essential in evaluating the success of a procedure and predicting outcomes in mitral valve repair surgery (MVR). Accurate assessment of MVA immediately after MVR is also necessary to assess the need for further mitral valve (MV) intervention, or conversion to MV replacement in cases of mitral valve stenosis (MS) or mitral valve insufficiency (MR).

Among the various echocardiographic techniques, the 2-dimensional (2D) planimetry technique and pressure half-time (PHT) technique have been widely used for peri-operative determination of the MVA. However, the 2D planimetry technique is not appropriate for measuring the MVA immediately after MVR, due to difficulties in optimizing the 2D plane of the TEE image, which must be perpendicular to MV orifice. The PHT technique is now in wide clinical usage for peri-operative evaluation of mitral valvular disease, because it is easy to perform and obtained during the entire diastolic phase, meaning that it reflects the haemodynamic states, although MVA determined by the PHT technique is not a gold standard technique for determination of MVA after MVR.

Real-time 3-dimensional (3D) TEE evaluation has become prominent for MV evaluation, giving a more exact and rapid understanding of the MV and surrounding structures. Several articles reported the efficacy of 3D TEE in the evaluation of the MVA and MV apparatus in MS or MR patients [1-8]. However, most research about the superiority of 3D TEE using the 3D planimetry technique has focused on pre-operative evaluations, and MVA determined by 3D TEE using 3D planimetry technique in the intra-operative post-MVR period has not been well researched. Although 3D planimetry technique shows more accurate MV orifice, compared with 2D planimetry technique [9], it is also measured at one point in time of MV maximal opening during diastolic phase without the haemodynamic states.

We hypothesized that MVAs with echocardiography, using intra-operative 3D planimetry versus intra- & post-operative PHT technique in MVR would be different.

The aim of the study was to evaluate the MVA determined by intra-operative 3D planimetry at post-MVR periods, compared with intra-operative TEE at post- MVR periods and post-operative transthoracic echocardiography (TTE) using PHT technique in patients undergoing MVR.

## Methods

### Clinical data

After a protocol review and approval by the Institutional Review Board of Konkuk University Medical Center, Seoul, South Korea [KUH1160044, (May, 2012)] and registration at http://cris.nih.go.kr (KCT0000447), medical records, including TEE and TTE data of patients who had elective MVR due to MS (>moderate) or MR (>moderate) in Konkuk University Medical Center from April 2011 to March 2012, were retrospectively reviewed for demographic, diagnostic, procedural and echocardiographic information. Patients with other concurrent cardiac valvular surgeries, reduced left or right ventricular function (ejection fraction <40%), or post-operative mitral valvular diseases (MS grade > moderate or MR grade > moderate at post-MVR period) were excluded.

Anaesthetic induction and maintenance were performed according to the standard institutional regimen with, remifentanil based anaesthesia with sevoflurane or propofol.

**Figure 1:**
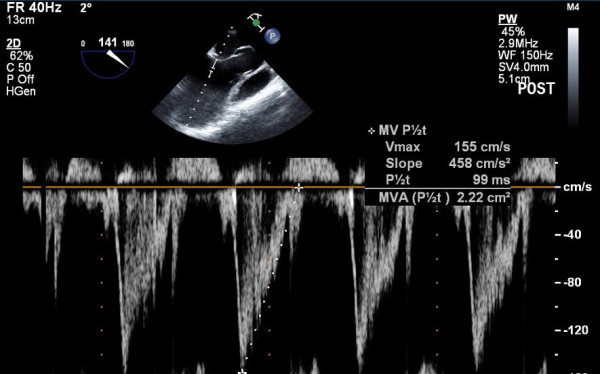
Determination of mitral valve area using pressure half-time with echocardiography.

**Figure 2:**
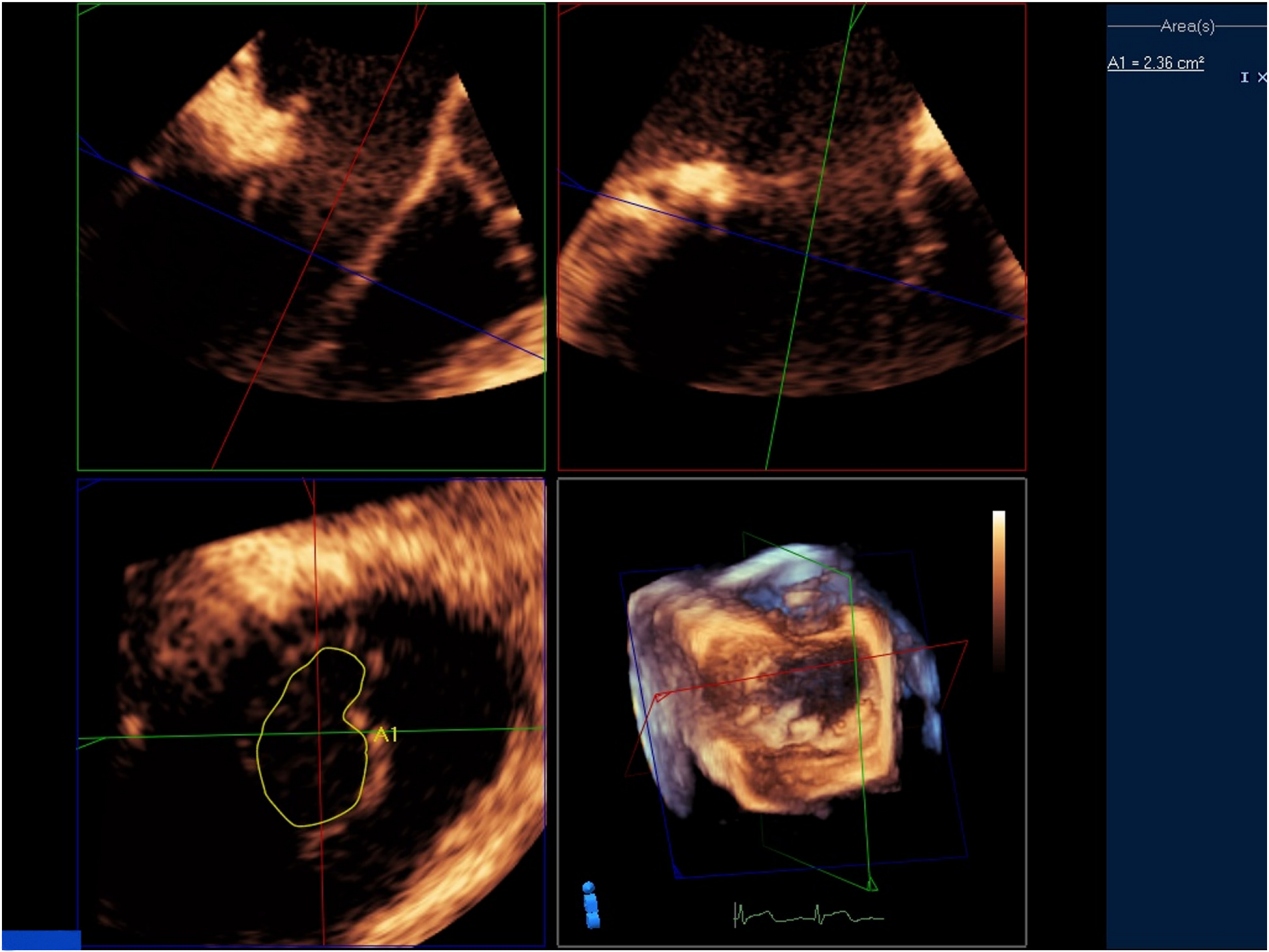
Determination of mitral valve area using 3-dimensional transoesophageal echocardiography.

After MVR, successful cardiopulmonary bypass (CPB) weaning and achieving stable haemodynamic state (mean arterial blood pressure > 60 mmHg, heart rate < 90 beats per minute, cardiac index > 2.0 l.min^-1^.m^-2^; approximately one hour after separation from CPB), MVA derived by PHT technique with TEE (MVA-PHT1) was determined. Three consecutive velocity-time integrals (VTI) of mitral inflow Doppler were traced by placing the sample volume of the pulsed-wave Doppler for MR or continuous-wave Doppler for MS on the tip of the MV leaflets in a mid-oesophageal aortic valve long-axis view. The PHT of the mitral inflow deceleration slope was determined from the stored mitral inflow Doppler VTI, and the MVA was then calculated using the following formula: MVA = 220/PHT (Figure 1) [10]. After acquisition of mitral inflow VTIs, MVA derived by 3D planimetry technique (MVA-3D1) was determined with 3D echocardiographic imaging platform (iE33; Philips Medical Systems, Andover, USA) and a 3D TEE probe (X-9; Philips Medical Systems). 3D full-volume images or 3D zoom images for an “*en face*” MV view from the left atrium (LA) or left ventricle (LV) perspective were acquired. The recorded 3D images were checked whether they contained whole structures of MV including anterior mitral leaflet (AML), posterior mitral leaflet (PML) and mitral valve annulus. If they did not contain the whole structures, the 3D images were re-acquired. The 2D image of the smallest MVA perpendicular to the mitral inflow at the maximal MV opening was acquired by aligning and cropping the acquired 3D images with suitable software (3DQ in Q-lab, Philips). Finally, the MVA was determined by circumferential tracing of the leaflet edges on the reconstructed MV 2D image (Figure 2). MVA-PHT1 and MVA-3D1 with TEE during intra-operative post-MVR period were measured by a cardiac anaesthesiologist. Two observers (A and B), who were blinded to the study and were cardiac anaesthesiologists, measured MVAs again with the PHT technique (MVA-PHT2 by observer A and MVA-PHT3 by observer B) and 3D planimetry technique (MVA-3D2 observer A and MVA-3D3 observer B), using same manners for MVA-PHT1 and MVA-3D1, from recorded intra-operative TEE data, to assess the inter-observer variability determination and the comparisons of MVA values. At post-operative day 7, the MVA using the PHT technique with TTE was measured by cardiologist in the same manner for MVA-PHT1 determination with parasternal apical view. Two observers, who were blinded to the study and were cardiologists, measured MVAs again with the PHT technique from recorded post-operative TTE data, to assess the inter-observer variability determination. MVA-TTE was defined as the mean of the recorded MVA using the PHT technique with TTE at post-operative day 7, and the MVAs measured by two cardiologists. All MVA measurements derived from intra-operative TEE data and post-operative TTE data were repeated 3 times and mean values were used for analysis.

### Surgical technique

For the final step of MVR, annuloplasty strip (Mitra-Lift strip, ScienCity, Inc., Seoul, South Korea) was applied.

For correction of MR, three technical manoeuvers are mainly applied; 1) lifting annuloplasty [11], 2) artificial chordae formation for anterior chordae problem and 3) patch valvuloplasty for posterior or lateral chordae problems [12].

For correction of MS, five technical manoeuvres are mainly applied; 1) lifting annuloplasty, 2) maximum commissurotomy, 3) anterior/posterior leaflet extension, 4) restoration of leaflets mobility by thinning and decalcification and 5) subvalvar procedure such as fenestration of fused chordae.

### Statistics

Based on preliminary medical records and echocardiographic data review of 10 MVR cases, the mean and standard deviation (SD) of MVA-PHT1 were 2.47 ± 0.57 cm^2^. The sample size of 26 was calculated to detect 20% difference (0.49 cm^2^) with a power of 0.80 and an α value of 0.05. Statistical analyses were performed with SPSS software (ver. 18.0; SPSS, Inc., Chicago, USA).

To assess inter-observer variability, the MVAs with the PHT and 3D planimetry techniques from TEE, and PHT technique from TTE data were analysed using intraclass correlation coefficient (ICC). Agreements between MVAs derived with PHT technique with TEE and those derived with 3D planimetry with TEE were assessed using the Bland-Altman method [13], and correlations between MVAs derived from the two techniques were evaluated with linear regression analysis.

Comparisons of MVA-PHT, MVA-3D and MVA-TTE were performed using One-way Analysis of Variance and pair-wise multiple comparisons were made using the Tukey method. Additionally, the agreements and correlations between MVA-TTE and the values of MVA derived by PHT technique and 3D planimetry technique with TEE were assessed by the Bland-Altman method and linear regression analysis. Data are expressed as the number of patients or means ± SD (95% confidence interval, CI). The null hypotheses of no difference were rejected if *p*-values were less than 0.05.

## Results

From April 2011 to March 2012, data for twenty-six of 107 patients’ medical records were analysed. Eighty-one patients were excluded for the following reasons: 57 for examinations only under 2D TEE platform without availability of 3D TEE, 19 for other concurrent valvular surgeries, 4 for low LV function (LV ejection fraction <40%), and 1 for MR grade > moderate at intra-operative post-MVR period. There were no patients with inadequate recorded 3D images for determination of MVA by 3D planimetry technique. Patients’ demographic data and pre-operative diagnosis are summarized in Table 1.

**Table 1:** **Patients’ demographic data and pre-operative diagnosis (*N*** **= 26)**

**Gender**	**(Male/Female)**	**12/14**
Age	(year old)	49 ± 16
Height	(cm)	164 ± 9
Weight	(kg)	61 ± 11
Diagnosis		
	MS	3
	MR	22
	Combined MS and MR	1

**Abbreviations**: MS, mitral valve stenosis; MR, mitral valve regurgitation.

LV ejection fraction of these patients by pre-operative TTE evaluation was 62.3 ± 9.6%. The pre-operative mean MVA in MS patients (*N* = 4) was 1.14 ± 0.32 cm^2^ indicating an above moderate degree of MS.

### Inter-observer variability for intra-operative MVA determination

The values of ICC were 0.90 with intra-operative PHT technique (MVA-PHT1, MVA-PHT2 and MVA-PHT3) and 0.78 with intra-operative 3D planimetry technique (MVA-3D1, MVA-3D2 and MVA-3D3).

**Figure 3:**
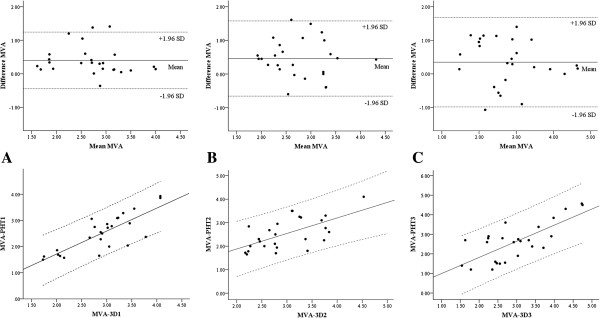
**Bland-Altman analysis and linear regression analysis between intra-operative mitral valve area measurement by 3-dimensional planimetry technique and pressure half-time technique.** (**A**) Analysis using recorded transoeosophageal echocardiographic data, Top: Bland-Altman plot, bias and precision = 0.40 ± 0.43 cm^2^, Bottom: linear regression analysis, *r* = 0.81 (*y* = − 0.04 + 0.88 · *x*, *p* < 0.001), (**B**) Analysis by observer **A** using intra-operative transoeosophageal echocardiographic data, Top: Bland-Altman plot, bias and precision = 0.46 ± 0.57 cm^2^, Bottom: linear regression analysis, *r* = 0.62 (*y* = 0.56 + 0.66 · *x*, *p* <0.001), (**C**) Analysis by observer **B** using intra-operative transoeosophageal echocardiographic data, Top: Bland-Altman plot, bias and precision = 0.35 ± 0.68 cm^2^, Bottom: linear regression analysis, *r* = 0.74 (*y* = 0.17 + 0.83 · *x*, *p* <0.001).

The biases (mean difference) and precisions (standard deviation of mean difference) between MVA-PHT and MVA-3D for agreement analysis were 0.40 ± 0.43 cm^2^ between MVA-PHT1 and MVA-3D1, 0.46 ± 0.57 cm^2^ between MVA-PHT2 and MVA-3D2, and 0.35 ± 0.68 cm^2^ between MVA-PHT3 and MVA-3D3, respectively. The correlation coefficients using linear regression analysis between MVA-PHT and MVA-3D were 0.81 (*y* = −0.04 + 0.88 · *x*, *p* < 0.001) for MVA-PHT1 and MVA-3D1, 0.62 (*y* = 0.56 + 0.66 · *x*, *p* <0.001) for MVA-PHT2 and MVA-3D2, and 0.74 (*y* = 0.17 + 0.83 · *x*, *p* <0.001) for MVA-PHT3 and MVA-3D3, respectively (Figure 3).

### Comparisons of MVA after MVR

The value of ICC was 0.91with post-operative MVA-TTE.

**Figure 4:**
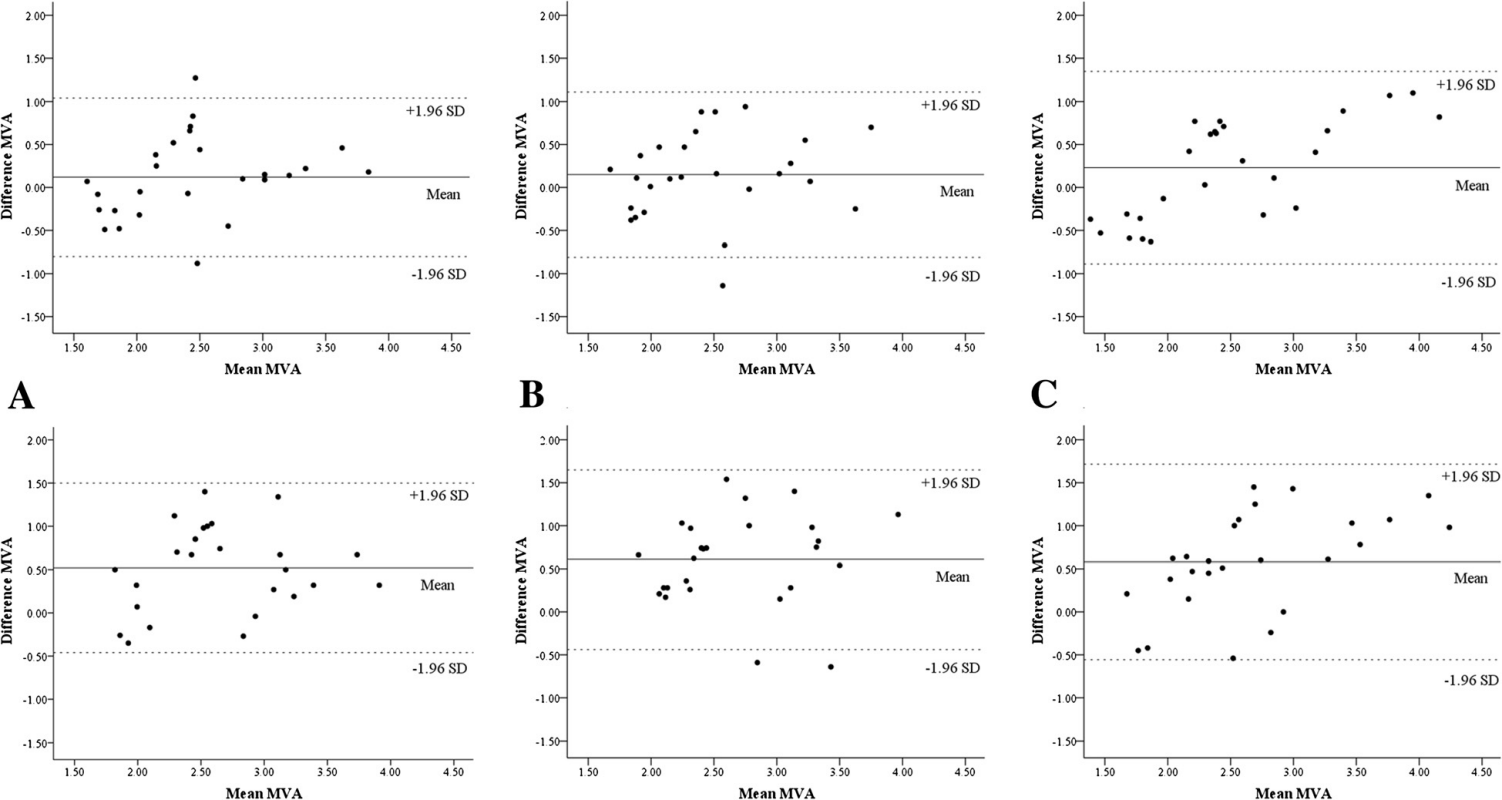
**Bland-Altman analysis between mitral valve areas derived by each measuring techniques.** (**A**) Top: Bland-Altman plot between MVA-PHT1 and MVA-TTE with bias and precision of 0.12 ± 0.47 cm^2^, Bottom: Bland-Altman plot between MVA-3D1 and MVA-TTE with bias and precision of 0.52 ± 0.50 cm^2^, (**B**) Top: Bland-Altman plot between MVA-PHT2 and MVA-TTE with bias and precision of 0.15 ± 0.49 cm^2^, Bottom: Bland-Altman plot between MVA-3D2 and MVA-TTE with bias and precision of 0.61 ± 0.53 cm^2^, (**C**) Top: Bland-Altman plot between MVA-PHT3 and MVA-TTE with bias and precision of 0.23 ± 0.57 cm^2^, Bottom: Bland-Altman plot between MVA-3D3 and MVA-TTE with bias and precision of 0.58 ± 0.58 cm^2^.

**Figure 5:**
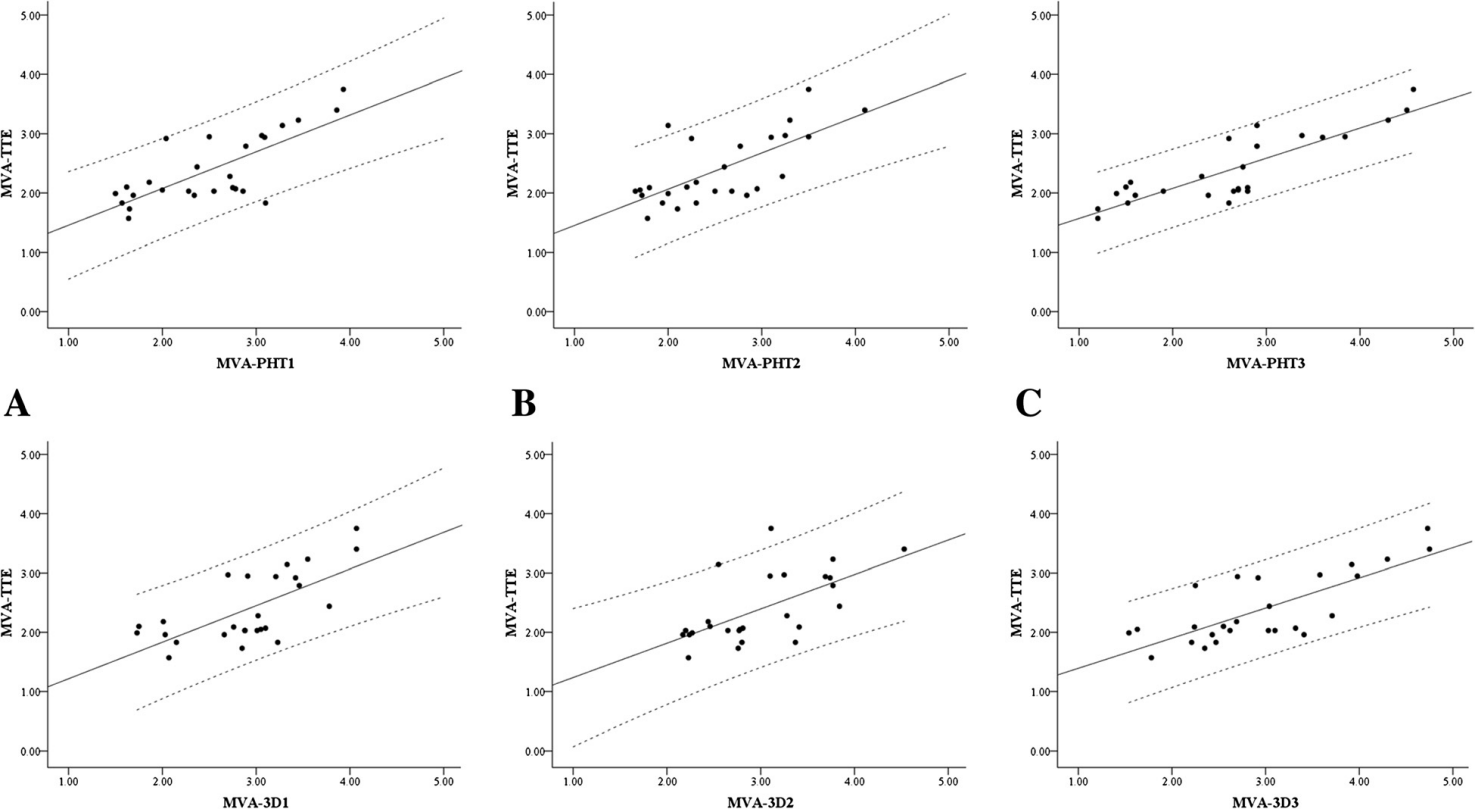
**Linear regression analysis between mitral valve areas derived by each measuring techniques.** (**A**) Top: linear regression analysis between MVA-PHT1 and MVA-TTE [*r* = 0.75 (*y* = 0.84 + 0.62 · *x*, *p* <0.001)], Bottom: linear regression analysis between MVA-3D1 and MVA-TTE [*r* = 0.68 (*y* = 0.60 + 0.62 · *x*, *p* <0.001)], (**B**) Top: linear regression analysis between MVA-PHT2 and MVA-TTE [*r* = 0.70 (*y* = 0.84 + 0.61 · *x*, *p* <0.001)], Bottom: linear regression analysis between MVA-3D2 and MVA-TTE [*r* = 0.62 (*y* = 0.66 + 0.85 · *x*, *p* <0.001)], (**C**) Top: linear regression analysis between MVA-PHT3 and MVA-TTE [*r* = 0.85 (*y* = 1.06 + 0.51 · *x*, *p* <0.001)], Bottom: linear regression analysis between MVA-3D3 and MVA-TTE [*r* = 0.76 (*y* = 0.88 + 0.51 · *x*, *p* <0.001)].

In comparisons of MVAs derived by each techniques, MVAs-3D [MVA-3D1 (2.91 ± 0.65 cm^2^), MVA-3D2 (3.00 ± 0.63 cm2) and MVA-3D3 (2.97 ± 0.88 cm^2^)] were larger than MVA-TTE (2.40 ± 0.59 cm^2^), and there were significant differences (*p* = 0.015, *p* = 0.003, *p* = 0.040, respectively; Table 2), although MVAs-PHT [MVA-PHT1 (2.51 ± 0.71 cm^2^), MVA-PHT2 (2.54 ± 0.67 cm2) and MVA-PHT3 (2.62 ± 0.98 cm^2^)] had no significant difference with MVA-TTE. The biases and precisions for echocardiographic records were 0.12 ± 0.47 cm^2^ between MVA-PHT1 and MVA-TTE, and 0.52 ± 0.50 cm^2^ between MVA-3D1 and MVA-TTE. The biases and precisions for observer A were 0.15 ± 0.49 cm^2^ between MVA-PHT2 and MVA-TTE, and 0.61 ± 0.53 cm^2^ between MVA-3D2 and MVA-TTE. The biases and precisions for observer B were 0.23 ± 0.57 cm^2^ between MVA-PHT3 and MVA-TTE, and 0.58 ± 0.58 cm^2^ between MVA-3D3 and MVA-TTE (Figure 4). The correlation coefficients for echocardiographic records were 0.76 (*y* = 0.52 + 0.73 · *x*, *p* <0.001) between MVA-PHT1 and MVA-TTE, and 0.68 (*y* = 0.60 + 0.62 · *x*, *p* <0.001) between MVA-3D1 and MVA-TTE. The correlation coefficients for observer A were 0.70 (*y* = 0.84 + 0.61 · *x*, *p* <0.001) between MVA-PHT2 and MVA-TTE, and 0.62 (*y* = 0.66 + 0.58 · *x*, *p* <0.001) between MVA-3D2 and MVA-TTE. The correlation coefficients for observer B were 0.85 (*y* = 1.06 + 0.51 · *x*, *p* <0.001) between MVA-PHT3 and MVA-TTE, and 0.76 (*y* = 0.88 + 0.51 · *x*, *p* <0.001) between MVA-3D3 and MVA-TTE (Figure 5).

**Table 2:** Mitral valve areas at intra- & post-operative different techniques

	**MVA-PHT (cm**^**2**^**)**	**MVA-3D (cm**^**2**^**)**	**MVA-TTE (cm**^**2**^**)**
Recorded data	2.51 ± 0.71 (2.23–2.80)	2.91 ± 0.65^*^ (2.65–3.17)	2.40 ± 0.59 (2.16–2.63)
Observer A	2.54 ± 0.67 (2.27–2.81)	3.00 ± 0.63^*^ (2.75–3.25)	
Observer B	2.62 ± 0.98 (2.22–3.02)	2.97 ± 0.88^*^ (2.61–3.33)	

Values are expressed as mean ± SD (95% confidence interval).

^*^: *p* < 0.05, vs. MVA-TTE.

**Abbreviations**: MVA, mitral valve area; PHT, pressure half-time technique using transoesophageal echocardiography at intra-operative post-mitral valve repair surgery period; 3D, 3 dimensional planimetry technique using transoesophageal echocardiography at intra-operative post-mitral valve repair surgery period; TTE, pressure half-time technique using transthoracic echocardiography at post-operative day 7.

## Discussion

The present study showed that MVA measured by 3D planimetry technique immediately after MVR was significantly different from MVA determined by PHT with TTE at post-operative day 7, in patients undergoing MVR. Tests of agreement showed that the intra-operative post-MVR 3D planimetry technique with TEE had larger biases and lower correlation coefficients than the intra-operative post-MVR PHT technique with TEE, compared with post-operative day 7 PHT technique with TTE as a reference value.

The PHT technique with TTE at post-operative day 7 was regarded as a reference value for MVA evaluation immediately after MVR period in the present study. Most of patients had already been discharged from the intensive care unit, and inotropics or/and vasopressors were stopped or tapered after postoperative day 7. Therefore, the effects of medications might be insignificant at post-operative 7 day and the patients’ haemodynamic conditions would be similar to the state of everyday life. Another reason for using the MVA derived by PHT technique with TTE at postoperative day 7 as reference value instead of that derived by 2D planimetry technique was to compare the MVAs measured by same technique, because our cardiac anaesthesiologist did not measure the MVA by 2D planimetry technique after CPB weaning due to difficulty to acquire proper 2D image.

Several studies reported the inaccuracy of PHT technique for MVA measurement, and the reasons such as geometric change of MV structures and net atrioventricular compliance when immediately after CPB ended in MVR [14-16]. And some studies suggested that other parameter such as pressure gradient was more useful for immediate postoperative MV evaluation to detect MS [17,18]. However, Maslow A *et al*. reported the importance of optimizing haemodynamics during assessment of MVA, and suggested that the PHT technique for MVA evaluation after MVR was still useful if haemodynamic optimization could be made [19,20]. In the present study, according to our institutional standard protocol, our cardiac anaesthesiologist maintained patients’ haemodynamic status stably within the predetermined range by using inotropics and vasopressors when echocardiographic examination was done after CPB weaning. And this haemodynamic status would be similar to the state of everyday life. Therefore, the values of MVA derived by PHT technique showed good correlation and were not different although those values were measured at different times.

In contrast to previous studies that reported the superiority and accuracy of 3D echocardiography in post-percutaneous balloon valvuloplasty and post-MVR period [4,21,22], the results of the present study suggest that 3D planimetry-derived MVA was larger, compared with MVA detected by post-operative PHT technique with TTE. The reasons may be elucidated from a review of the specific details of each technique. The repaired MV after MVR is still semilunar shape and all the edges of the AML usually cannot be included in the same image plane, especially at the lateral edges of the AML. If all edges of the AML are displayed clearly in one plane, the tip of the AML showing in the display may not be the real end of the AML. Although the 3D planimetry technique has the advantage of a more optimal 2D plane than the 2D planimetry technique for MV maximal opening through multiple orthogonal long-axis images of the 3D volume image, this limitation may influence the evaluation of MVA. Second, the flexible strip is used for annuloplasty in MVR. The material may influence the 3D TEE image as acoustic shadow and interrupt the detection of the exact PML tip. The interruption of the image may result in an inadequate angulation adjustment of the image plane and cause an inaccurate estimation of MVA. Third, measurement error by the observers may affect MVA. As MVA determination using the 2D planimetry technique [23,24], the shape and contrast of MV leaflet edges vary depending on the echocardiographic settings. For example, if the receiver gain setting is too low, the edges of the valve may be obscured, resulting in “echo dropout”, and the MVA will be overestimated. The opposite occurs when the gain settings are too high, with a falsely narrowed valve orifice. Additionally, the observer’s tendency of marginal tracing to detect MVA also may affect the values of MVA. In the present study, the intra-operative echocardiographic data was collected by a cardiac anaethesiologist. Two observers who did not know the recorded MVA values additionally measured the MVAs with the same recorded echocardiographic data, using the PHT and 3D planimetry techniques. The ICC values for the PHT technique and 3D planimetry technique with TEE were 0.90 and 0.78, respectively, and the ICC value for the PHT technique with TTE was 0.91. These values showed acceptable inter-observer variability. However, relatively lower ICC value of the 3D planimetry technique compared with that of the PHT technique means higher variability of 3D planimetry technique than PHT technique. And, this might be associated with above mentioned two reasons, namely the influence of the flexible strip to 3D image and the different observer’s tendency to detect the MVAs. Usually, the measurement of PHT was done by drawing a straight line from vertex along slope whereas the measurement of MVA by 3D planimetry technique was done by marginal tracing of AML and PML. Therefore, more complex measurement method than PHT technique might affect to the higher variablility of 3D planimetry technique.

The values of MVA derived by 3D planimetry technique at intra-operative post-MVR period were larger than those derived by PHT technique at post-operative period. Thus, it seemed that the MVA determination by 3D planimetry technique was inaccurate. As mentioned at introduction, the PHT technique is not yet the gold standard for measurement of MVA after MVR. The comparisons between MVAs derived by 3D planimetry and PHT techniques might be associated with difference of the measuring modality. Therefore, the assessment for accuracy of intra-operative post-MVR MVA measurement by 3D planimetry technique should be performed through comparison of the MVAs measured by the same technique with TTE, rather than PHT techniques. In the present study, 3D planimetry measurement of MVA at post-operative period could not be performed because there was no available 3D TTE platform. If it was possible to measure the MVA by 3D planimetry technique with TTE at post-operative period, the accuracy of intra-operative MVA by 3D planimetry with TEE could be assessed, and if intra-operative MVAs using PHT technique and 3D planimetry technique were compared with post-operative MVA derived by 3D planimetry technique with TTE, the result would be different.

The 3D planimetry technique is based on the same measurement concept for MVA by multi-detectors computed tomography (MDCT) because the evaluation is performed using planes in three corresponding perpendicular slice orientations. Lembcke *et al*. reported that MVA determined to be larger by MDCT than by the PHT technique [25]. They suggested the reasons of larger MVA by MDCT as follows: 1) anatomic MVA was determined by planimetry but effective MVA was determined by flow velocity and pressure gradient measurements. Therefore, planimetry technique and PHT technique represented two different parameters; 2) the haemodynamic effective orifice area was always smaller than the true actual geometric orifice area, because blood tended to flow through the center of the anatomic orifice; and, 3) as previously mentioned, the planimetry technique was measured at one time point of MV maximal opening during diastolic phase but the PHT technique was obtained during entire diastolic phase. The above reasons might be associated with the difference between MVAs by 3D planimetry technique and PHT technique in the present study. In other words, the reference values for MVA using the 3D planimetry technique would be different from that using the PHT technique after MVR.

## Conclusions

MVA measured by 3D planimetry technique with TEE at intra-operative post- MVR period was larger than that by PHT technique with TTE at post-operative period. However, it did not mean that the 3D planimetry technique was inaccurate but needs cautions at determination of MVA using different techniques.

## Abbreviations

MVA: Mitral valve area; TEE: Transoesophageal echocardiography; MVR: Mitral valve repair surgery; MV: Mitral valve; MS: Mitral valve stenosis; MR: Mitral valve insufficiency; 2D: 2-dimensional; PHT: Pressure half time; 3D: 3-dimensional; TTE: Transthoracic echocardiography; CPB: Cardiopulmonary bypass; MVA-PHT1: Mitral valve area measured as pressure half time technique with transoesophageal echocardiography; VTI: Velocity time integral; MVA-3D1: Mitral valve area measured as 3 dimensional planimetry technique; LA: Left atrium; LV: Left ventricle; AML: Anterior mitral leaflet; PML: Posterior mitral leaflet; MVA-PHT2: Mitral valve area measured as pressure half time technique with transoesophageal echocardiography by observer A; MVA-PHT3: Mitral valve area measured as pressure half time technique with transoesophageal echocardiography by observer B; MVA-3D2: Mitral valve area measured as 3 dimensional planimetry technique by observer A; MVA-3D3: Mitral valve area measured as 3 dimensional planimetry technique by observer B; MVA-TTE: Mitral valve area measured as pressure half time technique with transthoracic echocardiography; SD: Standard deviation; ICC: Intraclass correlation coefficient; CI: Confidence interval; MDCT: Multi-detectors computed tomography.

## Competing interests

The authors declare that they have no competing interests.

## Authors’ contributions

WS Kang participated in the design of study protocol, collection of data, statistical analysis of data and description of manuscript. JW Choi participated in the collection of data. JE Kang participated in the collection of data. JW Chung participated in the collection of data. SH Kim participated in the design of study protocol, collection of data, statistical analysis of data and description of manuscript. All authors read and approved the final manuscript.
